# Mechanical ventilation and the total artificial heart: optimal ventilator trigger to avoid post-operative autocycling - a case series and literature review

**DOI:** 10.1186/1749-8090-5-39

**Published:** 2010-05-17

**Authors:** Allen B Shoham, Bhavesh Patel, Francisco A Arabia, Michael J Murray

**Affiliations:** 1 Department of Anesthesiology, Mayo Clinic Arizona, 5777 East Mayo Boulevard, Phoenix, Arizona 85054, USA; 2 Department of Critical Care, Mayo Clinic Arizona, 5777 East Mayo Boulevard, Phoenix, Arizona 85054, USA; 3 Department of Cardiothoracic Surgery, Mayo Clinic Arizona, 5777 East Mayo Boulevard, Phoenix, Arizona 85054, USA

## Abstract

Many patients with end-stage cardiomyopathy are now being implanted with Total Artificial Hearts (TAHs). We have observed individual cases of post-operative mechanical ventilator autocycling with a flow trigger, and subsequent loss of autocycling after switching to a pressure trigger. These observations prompted us to do a retrospective review of all TAH devices placed at our institution between August 2007 and May 2009. We found that in the immediate post-operative period following TAH placement, autocycling was present in 50% (5/10) of cases. There was immediate cessation of autocycling in all patients after being changed from a flow trigger of 2 L/minute to a pressure trigger of 2 cm H_2_O. The autocycling group was found to have significantly higher CVP values than the non-autocycling group (P = 0.012). Our data suggest that mechanical ventilator autocycling may be resolved or prevented by the use of a pressure trigger rather than a flow trigger setting in patients with TAHs who require mechanical ventilation.

## Background

In 2006, end-stage cardiomyopathy was the primary cause of death for almost 60,000 Americans[1]. Transplantation would have prevented many of these deaths; however, only 3205 patients *worldwide *received transplanted hearts in 2006[2]. Since the publication of the REMATCH study, [3] patients with end-stage cardiomyopathy have increasingly received total artificial hearts (TAHs) as a bridge to cadaveric cardiac transplantation, with surgeons currently implanting third-generation devices[4]. We must be diligent in maintaining our knowledge of these devices, as well as our skills in providing care to patients with TAHs.

**Figure 1:**
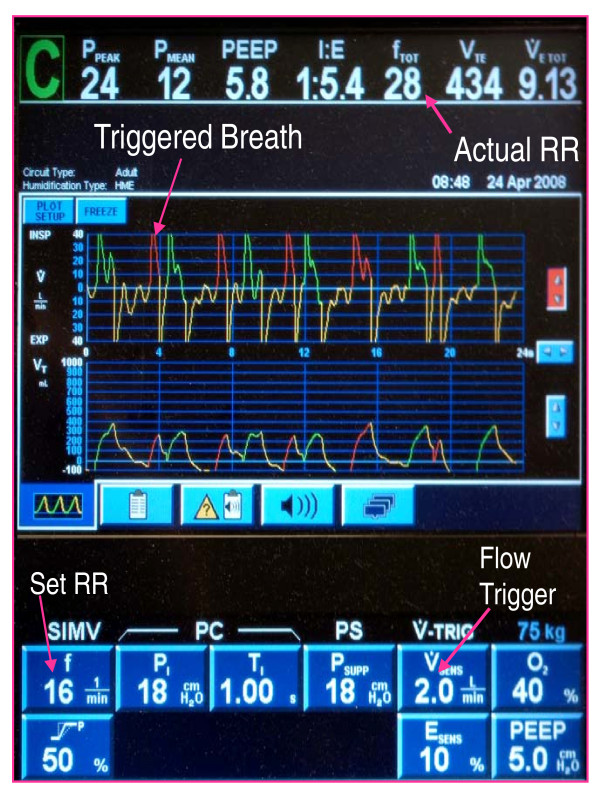
**TAH induced autocycling is present with an actual RR of 28 using a flow trigger**.

**Figure 2:**
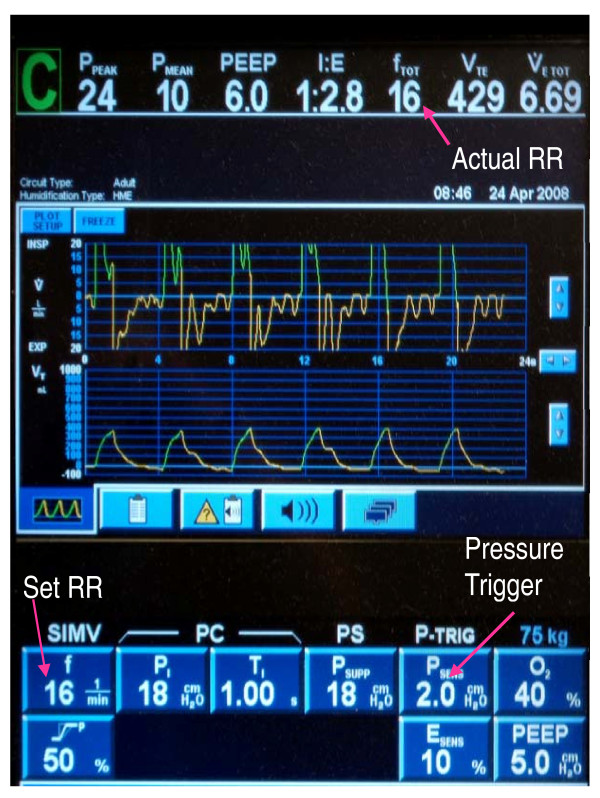
**Autocycling is absent with the use of a pressure trigger as the actual RR = set RR of 16**.

After they have had a TAH implanted, patients typically remain tracheally intubated and mechanically ventilated for several days to weeks. While caring for such patients in our institution, we noticed that many developed postoperative autocycling of the mechanical ventilator when a flow trigger was used to initiate breaths (Figure 1), which subsequently stopped when we switched the ventilator to a pressure trigger (Figure 2). Autocycling refers to the inappropriate triggering of a ventilator assisted breath in the absence of a spontaneous patient effort. These observations piqued our curiosity regarding the frequency of this occurrence and precipitated the initiation of this retrospective chart review.

## Methods

Following institutional review board approval, we reviewed the computerized medical records of all patients with a TAH placed at our institution between August 2007 and May 2009. We created a database using Excel (Microsoft, Redmond, WA) and recorded the central venous pressure (CVP) while the patients were mechanically ventilated, the positive end expiratory pressure (PEEP), mode of ventilation, body mass index (BMI), TAH rate, and both the set and actual respiratory rate while the patients' ventilators were set for both flow and pressure triggering. A 1-tailed equal variance student *t *test was done to compare the BMI and CVP values of the autocycling group and the non-autocycling group.

The medical records of 10 patients were identified for review (Table 1). All patients were older than 40 years of age; exact ages have not been included in this report because of the need for patient confidentiality. The artificial heart device used in all patients was the SynCardia CardioWest (Tucson, AZ). The mechanical ventilator used in all patients was the Puritan Bennett 840 (Pleasanton, CA).

**Table 1:** Case Series Data

Patient	Mode of ventilation	CVP	BMI	RR^actual/set ^with flow trigger	RR^actual/set ^with pressure trigger	TAH rate	PEEP
Autocycling group							

1	ACV	20	27.5	37/24	26/26	101	5

2	SIMV + PS	21	28.7	38/6	10/10	123	5

3	SIMV + PS	22	39.7	16/12	18/18	115	6

4	SIMV + PS	20	25.9	37/12	12/12	131	5

5	SIMV + PS	19	23.6	27/12	12/12	115	16

Nonautocycling group							

6	SIMV + PS	18	31.2		10/10	110	8

7	SIMV + PS	7	32.3	18/18	30/30	110	25

8	SIMV + PS	15	32.3	10/10		125	5

9	SIMV + PS	19	25.8	18/12	18/12	120	5

10	SIMV + PS	10	30.8	14/10	12/10	110	5

ACV refers to assist control ventilation; SIMV, synchronized intermittent mechanical ventilation; PS, pressure support; CVP, central venous pressure; BMI, body mass index; RR, respiratory rate; TAH, total artificial heart; PEEP, positive end expiratory pressure.

## Results

Data from all 10 patients (Table 1) shows that 8 had a flow trigger of 2 L/minute as the initial ventilator setting, with a change in all 8 to a pressure trigger of 2 cm H_2_O within the first 48 hours after TAH placement. One patient was kept on a flow trigger of 2 L/minute until extubation without any autocycling noted, and another patient was kept on a pressure trigger of 2 cm H_2_O until extubation without any autocycling recorded.

Five of the 10 patients developed autocycling of the ventilator, but autocycling ceased immediately in all of these patients when their ventilator settings were changed from a flow trigger of 2 L/minute to a pressure trigger of 2 cm H_2_O. There were no documented changes in the patients' levels of sedation, mentation, or neuromuscular blockade when these changes were made.

## Discussion

A search of PubMed using the terms *ventilation *and *artificial heart *and *autocycling *and *artificial heart *on May 23, 2009, did not reveal any publications regarding autocycling associated with TAH devices, nor did we find a description of optimal mechanical ventilator settings for patients who have received a TAH or strategies to postoperatively manage the ventilators of such patients.

Potential consequences of mechanical ventilator autocycling include respiratory alkalosis, barotrauma, patient ventilator dysynchrony, and over use of sedative medications[5,6]. In 1 of our patients, the presence of autocycling resulted in evaluation and work-up for central hyperventilation syndrome. Such an evaluation typically includes invasive testing that could be potentially harmful to the patient.

Flow- and pressure-triggered mechanical ventilator modes are designed to allow and assist with spontaneous ventilation. In a pressure-trigger mode, the patient's inspiratory effort is recognized when the airway pressure decreases below the baseline level of PEEP by the set trigger sensitivity (2 cm H_2_O in our cases). Once this occurs, the ventilator delivers an assisted breath.

In a flow-trigger mode using the Puritan Bennett 840 mechanical ventilator, the baseline continuous expiratory flow is set at 1.5 L/min greater than the set flow trigger (2 L/min in our case), resulting in a continuous expiratory flow of 3.5 L/min. The patients' inspiratory effort is recognized as a drop in expiratory flow by the set trigger sensitivity, consequently resulting in a ventilator-assisted breath. In our case, this would occur when expiratory flow is less than 1.5 L/min.

Use of a flow trigger has been shown to decrease the inspiratory work of breathing in patients with chronic obstructive pulmonary disease and intrinsic PEEP (iPEEP)[7]. In the case of iPEEP, the patient would have to produce a greater negative pressure to overcome the difference between intrinsic and circuit PEEP. However, with newer ventilator programming, the inspiratory work of breathing is similar between flow and pressure trigger modes[8,9].

Any device that alters resistance from the alveolus to the sensor at the y-piece, such as gas leaks from ventilator circuits, leaks in the cuff of the tracheal tube[5,10]. a heat moisture exchanger, [6] and an in-line catheter, can be a source of ventilator autocycling[11]. Cardiac oscillations are another well-known source of autocycling and have been described in patients in the ICU and during general anesthesia[12]. Cardiac oscillations leading to autocycling in patients who have undergone cardiac surgical procedures has been shown to be relatively common with flow-trigger settings, particularly in patients who have large cardiac outputs, large heart size, low respiratory system resistance, and an elevated CVP[13]. The differences we have observed in incidence of autocycling may not only reflect the method of triggering (flow vs. pressure), but also the sensitivity of the trigger used as the more sensitive the setting, the more likely autocycling will occur; flow-triggering has been shown to be particularly sensitive to circuit leaks[5,6,10,11].

The autocycling group in our case series did have significantly higher CVP values than the non-autocycling group (P = 0.012), though we can not draw any causative conclusions with this post hoc data. An elevated CVP may reflect decreased intra-thoracic compliance, thereby increasing transmitted pressure changes to the airway with resultant autocycling. The elevated CVP may also simply be a consequence of mechanical ventilator autocycling rather than a cause.

TAH oscillations induce significant pulmonary volume displacement as there are large pneumatic pressure changes for each beat[14,15]. A patient with post-operative respiratory failure after Jarvik-7 TAH placement showed significant lung displacement during apnea[15]. In fact, this patient's cardiac oscillations were large enough for sustained alveolar ventilation with an arterial pCO_2 _of 61 after one hour of total apnea. The Jarvik-7 TAH is the most recent structural cousin to our current CardioWest TAH device.

Why is it that autocycling occurred in 50% of our patients with a flow trigger but not with a pressure trigger? Modern mechanical ventilators maintain PEEP and compensate for changes in circuit pressure by adjusting the exhalation valve with an active microprocessor control throughout the expiratory period[16]. The microprocessor actively adjusts the expiratory valve to maintain a set PEEP, ultimately leading to subsequent changes in circuit flow. The result is that pressure is maintained at the expense of a change in flow. The CardioWest TAH initiates very large intra-thoracic pressure changes that, by definition, are transmitted to the airway. With a pressure trigger, PEEP maintenance may compensate for the TAH-induced pressure changes prior to a breath being triggered. With a flow trigger the microprocessor once again compensates for the pressure change induced by the cycling of the TAH. This compensation, leads to pressure maintenance at the expense of a change in flow, which may then trigger an autocycled breath if timed correctly.

## Conclusion

In summary, autocycling of the mechanical ventilator occurred in 50% of patients who had received TAHs with the use of a flow trigger ventilator setting. Autocycling was resolved in all these patients by changing from a flow trigger to a pressure trigger ventilator setting. Mechanical ventilator PEEP maintenance maintains pressure at the expense of altered flow, ultimately leading to autocycling in the case of a flow trigger. Given the frequency of autocycling in the ICU, this information may be applicable to other patients who are mechanically ventilated. Because advanced ventilator software has significantly diminished differences in inspiratory work of breathing, physicians may consider using a pressure trigger as an initial ventilator mode, or switching to this mode in patients suspected of, or at high risk for autocycling.

## Competing interests

The authors declare that they have no competing interests.

## Authors' contributions

AS reviewed the literature, drafted and completed the manuscript. BP and MM assisted in drafting and reviewing the manuscript. FA performed surgical interventions and reviewed the manuscript. Both FA and BP participated in post-operative management of patients studied. All authors read and approved the final manuscript.
